# Spatial indices quantifying exposure to swine farming in North Carolina

**DOI:** 10.3389/fvets.2025.1552028

**Published:** 2025-04-30

**Authors:** Kaushi S. T. Kanankege, Rashmi Kandwal, Andres M. Perez

**Affiliations:** ^1^Department of Veterinary Population Medicine, College of Veterinary Medicine, University of Minnesota, St Paul, MN,United States; ^2^Independent Researcher, Minneapolis, MN, United States

**Keywords:** environmental epidemiology, CAFO, animal agriculture, geostatistcs, One Health

## Abstract

**Introduction:**

Proximity to swine farms is often used as a surrogate in exposure assessments, allowing for the relative quantification of potential pollutant dispersion, odor intensity, and health impacts on neighboring communities. However, defining exposure is complex, and the resulting risk profiles can vary depending on the definition used.

**Methods:**

To quantify the spatially based exposure of surrounding communities to swine farms in North Carolina, three spatially explicit metrics were developed at the census tract-level: IDx1: number of households within 1-mile from a hog farm, IDx2: Co-kriging using the number of hogs and manure lagoons, and IDx3: hog density per square mile. Then, the correlation between these indices and Centers for Disease Control and Prevention (CDC)‘s Social Vulnerability Index (SVI) and Environmental Justice Index (EJI), which are generalized vulnerability measures, was evaluated to assess direct impact from swine farms versus multiple stressors.

**Results:**

The three indices differed visually, with IDx3 strongly correlated with IDx1 (0.8) and moderately correlated with IDx2 (0.4). CDC EJI and SVI were not prominently correlated with any of the swine-farm specific indices (≤0.3) indicating limited overlap. The correlation between swine-farm-specific indices and CDC SVI was slightly pronounced in rural areas indicating socially vulnerable populations are more likely to live near swine farming areas in rural census tracts. Having swine farm-specific indices offers a more tailored and nuanced understanding of the potential health and environmental risks. However, the differences between the maps and the varying correlations underscored how different definitions of exposure can yield distinct narratives about which neighborhoods are at risk. Defining and measuring potential exposure, considering factors like proximity, duration, frequency, vulnerability, and cumulative impact, is highly challenging.

**Discussion:**

The study emphasizes the need for a hierarchical framework to quantify and compare environmental exposures, addressing risk-modifying factors and individual-level exposure across space and time before implying direct exposure risks. This approach enables more informed planning for targeted solutions and fosters collaboration among stakeholders, facilitating critical discussions on integrated One Health solutions.

## Highlights

- Three spatial indices developed to quantify exposure to nearby swine farms.
- Indices account for animal count, manure lagoons, and distance to addresses.
- Created at census tract level for comparison with CDC’s SVI and EJI indices.
- Highlights the challenge of defining exposure, which can be measured in various ways.
- Facilitates comparison of swine farm exposures to multi-stressor exposure indices.

## Introduction

The intersection of animal agriculture, human, and environmental health often sparks intense debates. The main challenge is how to feed the growing population while minimizing harm to the planet and its people, animals, and their shared environment. Achieving this balance involves understanding exposure factors, reducing environmental risks for vulnerable communities, and co-creating pathways for promoting sustainable agricultural goals (1). Often in the view of environmental and public health there is an implicit bias toward animal agriculture where it is villainized yet often neglected (2, 3). This leaves producers seeking political or financial protection, as available solutions are limited and lack sufficient funding or technical support. The absence of evidence-based, scalable solutions that balance economic feasibility with environmental and health objectives hinders the animal farming industry’s sustainable transition.

Proposing systemic and long-term changes to the U.S. farming model requires evidence-based scalable long-term solutions that consider social, demographic, geographical, and environmental factors. The growing interest in sustainable agriculture that can ensure the health of the environment, and the residents holds a substantial importance under translational research. Environmental exposures are not homogeneously distributed across populations, with social determinants of health and natural barriers, pollutant behavior, and local conditions significantly shaping geographic disparities (4, 5). The way exposure is defined and measured can yield diverse interpretations for the same source of pollutant or the environmental stressor. Explicitly defining exposure risk in terms of space–time and dose–response to accurately gage its impact is challenging, particularly in the absence of robust empirical and systematic evidence (6, 45). For example, U.S. Centers for Disease Control and Prevention (CDC) have already developed social vulnerability (SVI) and environmental justice (EJI) indices at the census tract level for the whole United States (5, 7), (22, 23), however, the absence of a swine farm specific indicator within the EJI poses significant limitations not only for assessing the impact of livestock farming on community health and environmental equity; but also, for livestock farming communities understand the specific and direct impacts.

Our objective was to develop swine farm-specific exposure indices, which are tools for quantifying spatial exposure pathways, building on our previous research in North Carolina (8). The discord between communities residing near swine farms and the agricultural communities, characterized by frequent legal disputes and protests underscores the necessity of scrutinizing the specific context in North Carolina (9, 10). The indices were developed as a function of swine density, the number of swine farms and manure lagoons, and the distance from farms to the residential or commercial addresses within each census tract. While individual-level exposure measures are imperative to conclude exposure impact on an individual’s health, we trust that these indices provide a foundation for planning future exposure studies, comparing swine farming-specific risks with other environmental stressors, developing a hierarchical framework to rank influential stressors within a census tract, and guiding decisions to promote sustainable agriculture, environmental protection, and community health.

## Methods and data

### Study area

The U.S. southeastern state of North Carolina (Latitude: 35^o^N, Longitude: 79^o^W) inhabits over 10 million people, with 12.8% of them living below poverty (i.e., 11th lowest household income rank among the 50 U.S. states) (11). The state has a range of urban and rural forms with 33.9% of the population classified as rural, compared to 19.3% of the urban population, reflecting different facets of vulnerabilities in urban and rural settings (12). Raleigh and Charlotte, located in the central and western parts of the state, are the largest metropolitan areas. Rural parts of North Carolina, especially in the mountains and in the east, are affected by persistent inter-generational poverty (13–15). North Carolina is a major swine farming state in the United States, with more than 60% of its 8.9 million hogs concentrated in the southeastern region (16). The swine feeding operations in North Carolina are required to obtain a permit from North Carolina Department of Environmental Quality confirming the swine waste management system complies with the state requirements (17, 18).

### Data sources

The following spatial and non-spatial datasets were used in the analysis. Spatial base, i.e., the GIS shapefile of North Carolina state borders, census tracts for 2020 (*n* = 2,672), urban and rural census tract categorization of 2020, and address points of 2019 were downloaded from NCOneMap geospatial data website^1^ (46, 47). Swine farm (*n* = 2,072) data, including farm location, animal counts allowed, and the number of manure lagoons were extracted from the North Carolina Department of Environmental Quality (DEQ) website in June 2023^2^ (19–21). Overall, 9,612,252 hogs were allowed in the registered swine farms in the state. While the hog operations in North Carolina were defined as “any agricultural feedlot activity involving 250 or more swine with a waste management system, or any agricultural feedlot activity with a liquid animal waste management system that discharges to the surface waters of the state”; the data included farms with allowable number of animals ranging from 18 to 64,680 hogs with an average of 4,278 hogs and median of 3,500 hogs per operation.

CDC SVI indicates the relative vulnerability of U.S. Census tract in responding to and recovering from public health emergencies, based on 14 factors that represent four themes: (1) Socioeconomic, (2) Household composition/disability, (3) Minority status/language, and (4) Housing type/transportation. The overall tract summary ranking variable (RPL_THEMES) from year 2022 (*n* = 2,672) was used in the analysis (7, 22). Similarly, the Environmental Justice Index (EJI) utilizes data from various U.S. agencies, including the Census Bureau, EPA, Mine Safety and Health Administration, and CDC, to evaluate the cumulative impact of environmental injustice on health in each census tract (5, 22). The EJI ranking is based on 36 factors spanning environmental, social, and health dimensions. The EJI classifies these factors into three modules and 10 domains, encompassing indicators such as minority status, socioeconomic factors, air and water pollution, hazardous sites, and pre-existing chronic disease burdens. However, the 2022 EJI estimates do not particularly include the number of swine farms per census tract or a comparable variable, making this comparison more valuable for assessing the effects of other environmental challenges relative to those posed by swine farms themselves. For this study, we used the 2022 EJI values, developed at the census tract level (*n* = 2,192 tracts) (5), (CDC EJI, 2022). Specifically, the RPL_SER variable of CDC EJI, which is the percentile ranking of the summations of environmental burden and social vulnerability modules, was used.

### Spatial indices representing exposure to swine farming areas in North Carolina

#### Index 1: addresses within 1-mile buffer from a swine farm

Assuming that the distance from a farm to an address represents the severity of exposure, the count of unique addresses within 1-mile from a farm per census tract was calculated (i.e., Addresses within 1 mile of any swine farm in the census tract were counted, without duplication for those within 1 mile of multiple farms). Unique parcel addresses were used without distinguishing between residential, commercial, and other types of addresses, as this level of detail was not available.

The index value of higher number means multiple households are within the range of 1-mile exposure in the census tract. While 1-mile is an arbitrary distance, research investigating health effects of environmental exposure have often utilized 1-mile buffer zones around the pollution source in their analysis (23, 24). ArcGIS PostgreSQL Version 12.4 was used to create buffer zones and count the number of houses per buffer area. The concept of buffer zones is often used to conduct prevention and control plans related to disease spread between animal farms as well as in relation to determining protective zones such as riparian buffer zones which is the vegetated area adjacent to a water body that helps protect water quality by filtering pollutants, stabilizing banks, and supporting biodiversity (25, 26). Using a 1-mile buffer to determine risk is simple, comparable, and feasible, serving as a basic proxy for proximity-based exposure, making it informative for stakeholders and policymakers. However, it oversimplifies dynamic factors, assumes uniform risk, and may misclassify affected populations, making it less accurate without additional context such as wind direction, topography, or pollutant dispersion patterns in relation to natural barriers, vegetation, soil, water, and air features.

#### Index 2: co-kriging using number of pigs and manure lagoons

The Swine farm locations, number of animals, and number of manure lagoons were used to generate a spatially continuous variable using co-kriging. Kriging is a geostatistical interpolation method used to estimate values at unmeasured locations based on the spatial autocorrelation of observed data points (27). It considers both the distance and the degree of variation between known data points to produce a statistically optimized surface. Co-kriging interpolates and estimates the values at unsampled locations using two or three spatially correlated variables, and this geostatistical modeling technique creates a continuous surface (27, 28). Here the number of manure lagoons and the log of the number of animals allowed was used in the co-kriging model. Since the number of animals allowed per site ranges from 18 to 64,480, and the number of lagoons ranges from 0 to 13, applying the logarithm of the animal count helped create a more comparable scale between the two variables. This transformation reduces the skew caused by the large variation in animal counts and ensures that both variables contribute similarly to the model, improving the overall fit and interpretation of the results.

In this model, we assumed that the ‘exposure’ is a continuous variable applicable equally to all directions from a farm, regardless of the wind direction, terrain characteristics, or surface run-off. In general, the major swine farming areas in North Carolina have low runoff with <10% runoff percentile^3^ (29, 48). Moreover, while water soluble pollutants such as nitrates can still leach into groundwater, soils with low runoff such as sandy soil allow precipitation to filter through the soil before mixing with groundwater. The maximum value of co-kriged surface was extracted for each census tract and standardized as a percentage (i.e., The maximum values from the co-kriged surface were summed across the entire area, and each census tract’s value was then expressed as a percentage of the total sum). Co-Kriging produces a continuous surface, resulting in multiple values within a census tract. Values are higher near swine farms and lower farther away. To capture the “worst-case scenario,” the maximum co-kriging value was extracted for each census tract. In essence, this index represents areas where multiple large swine farms with multiple manure lagoons are clustered closer to each other, thereby increasing potential exposure.

Kriging offers precise spatial predictions by incorporating data correlations and creating smooth, continuous exposure maps, making it more flexible and accurate than fixed-distance methods like buffers. It is particularly effective in visualizing risk gradients and adapting to environmental variability. However, it is a data-intensive, computationally complex geostatistical technique. Additionally, interpolation errors and reliance on assumptions about spatial correlations can introduce uncertainty, especially with sparse or unevenly distributed data. The DEQ database for swine farms and manure lagoons in North Carolina is well-established and consistently maintained, making its records of permitted animal numbers and registered manure lagoons a reliable representation of swine farming operations in the state (see text footnote 2). The Kriging/co-kriging model function of the Geostatistical Analyst Wizard of ArcMap 10.7 was used to perform the analysis and creating an interpolation map.

Co-kriging accounts for the spatial interdependence among swine farms, making it a valuable indicator for broad exposures such as the land application of manure through aerial spraying, assuming that manure from lagoons is likely sprayed onto the nearest crop agricultural areas. Similar approaches have previously been used in North Carolina to quantify hog farming- related exposure in air pollution modeling (30).

#### Index 3: swine density per square mile

In this relatively simpler index, the number of pigs per square mile was calculated per census tract. In this context, more animals in the areas with many swine farms closer to each other represent higher exposure compared to areas where farms that have a smaller number of pigs and are far apart. Previous studies have conducted this hog density approach to measure exposure (3, 8). Using swine density allows for precise, scalable assessments of livestock concentration, facilitating comparisons and integration with other risk factors. However, this method assumes uniform distribution of the farms throughout the census tract, which may not be accurate if they are in one corner or clustered, thereby overlooking land use heterogeneity.

### Correlation analysis

Spearman correlations were calculated between the three indices and the CDC’s SVI and EJI using the R statistical software base and the ‘PerformanceAnalytics’ package (31, 32). As recommended by the CDC SVI and EJI documentation, one variable was extracted to indicate the overall summary ranking representing the socioeconomic status, household characteristics, racial and ethnic minority status, and housing type and transportation (variable name in the database: RPL_THEMES). Similarly, the variable extracted from CDC EJI was named ‘RPL_SER’ in the database which is the indicator of environmental justice excluding the accounting for health variables. The RPL_SER represents rankings for the entire USA, and not specific to individual states. However, for comparative purposes, such as calculating correlations between variables—the relative RPL_SER value was considered a sufficient as the scaling still reflects variation across census tracts. Correlation coefficient ≥0.1 were considered toward the analysis. Further analysis was conducted to compare the changes of these correlations at urban (*n* = 2,000) and rural (*n* = 672) census tracts; specifically, census tracts with at least 50% of their land area within city limits were classified as urban. Of the 53,812 square miles of area including water in North Carolina, only 3,058 square miles (6%) were within the city limits considered as urban areas. The urban census tracts had 878 swine farms with 4,021,731 (42%) allowable animals in them, whereas the rural ones had 1,194 farms with 5,590,521 (58%) animals allowable.

## Results

### Spatial indices representing exposure to swine farming

#### Index 1: addresses within 1-mile buffer from a swine farm

**Figure 1:**
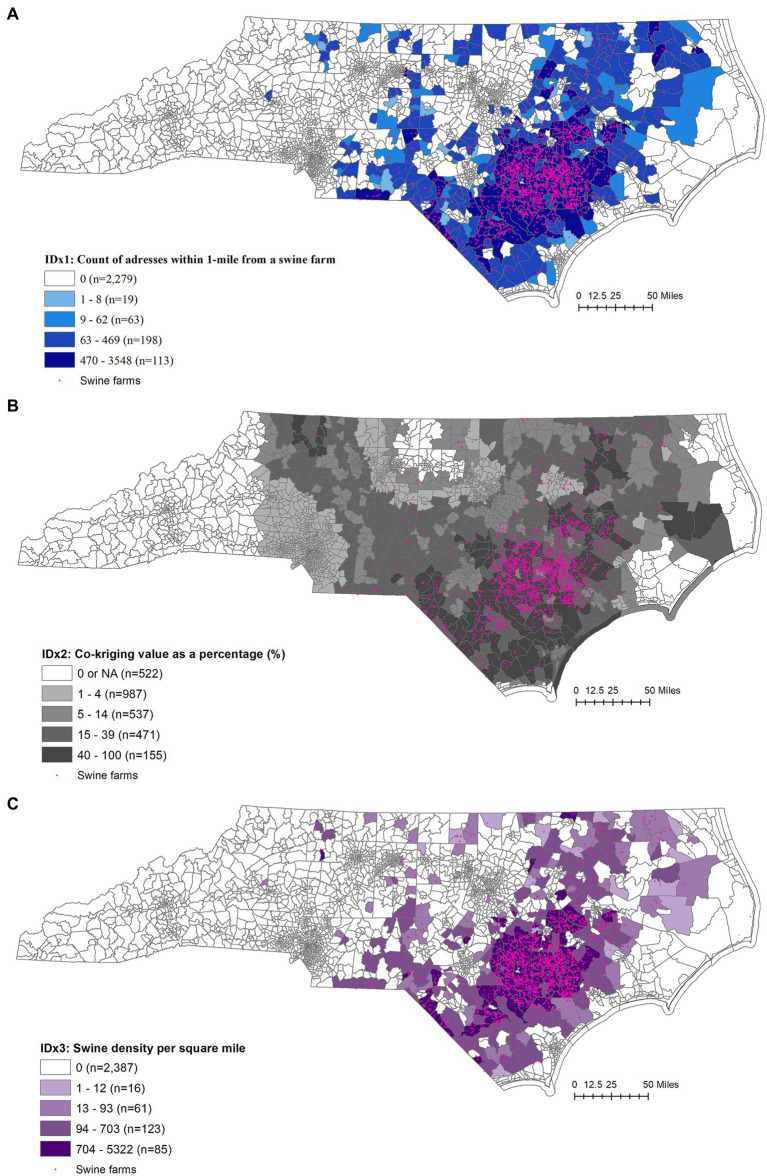
**(A)** Index 1: addresses within 1-mile buffer from a swine farm, depicting the number of addresses within a 1-mile buffer zone from a swine farm in each census tract. The index was categorized based on geometric intervals. The number of census tracts in each category is provided in parentheses. Category 1 represents zero or no addresses within a 1-mile radius from a swine farm in that census tract. **(B)** Index 2: co-kriging using number of pigs and manure lagoons, where co-kriging value is rescaled as a percentage of all values within the interpolated area of North Carolina, depicted at census tract level. The index was categorized based on geometric intervals. The number of census tracts in each category is provided in parentheses. **(C)** Index 3: swine density per square mile. Shown at the census tract, swine density was calculated based on the allowable number of pigs and hogs per farm, as registered at the North Carolina Department of Environmental Quality website in 2022 (https://deq.nc.gov/), (20). The index was categorized based on geometric intervals. The number of census tracts in each category is provided in parentheses.

As seen in Figure 1A, we observe that in areas with high swine farm density, like Duplin County, North Carolina, the number of households within a 1-mile buffer is generally high. However, across different census tracts, the number of unique addresses within the buffer zone varies significantly, ranging from 0 to 3,548. The mean value is 66.3 addresses, with a standard deviation of 278.2.

#### Index 2: co-kriging using number of pigs and manure lagoons

Number of manure lagoons and the log of the number of animals allowed was used in the co-kriging model. The co-kriging index value for census tract ranged between 5.1–9.3 raw values, the index was represented as a percentage for value of the interpolated area of North Carolina resulting in mean value of 9.0% with a std. deviation 17.0% (Figure 1B). All parameter settings that were used in the co-kriging are summarized in Supplementary Table S1.

#### Index 3: swine density per square mile

The hog density per square mile per census tract ranged between 0 and 5,322 pigs per square mile with the mean of 69 and std. dev of 351 pigs per square mile (Figure 1C).

**Figure 2:**
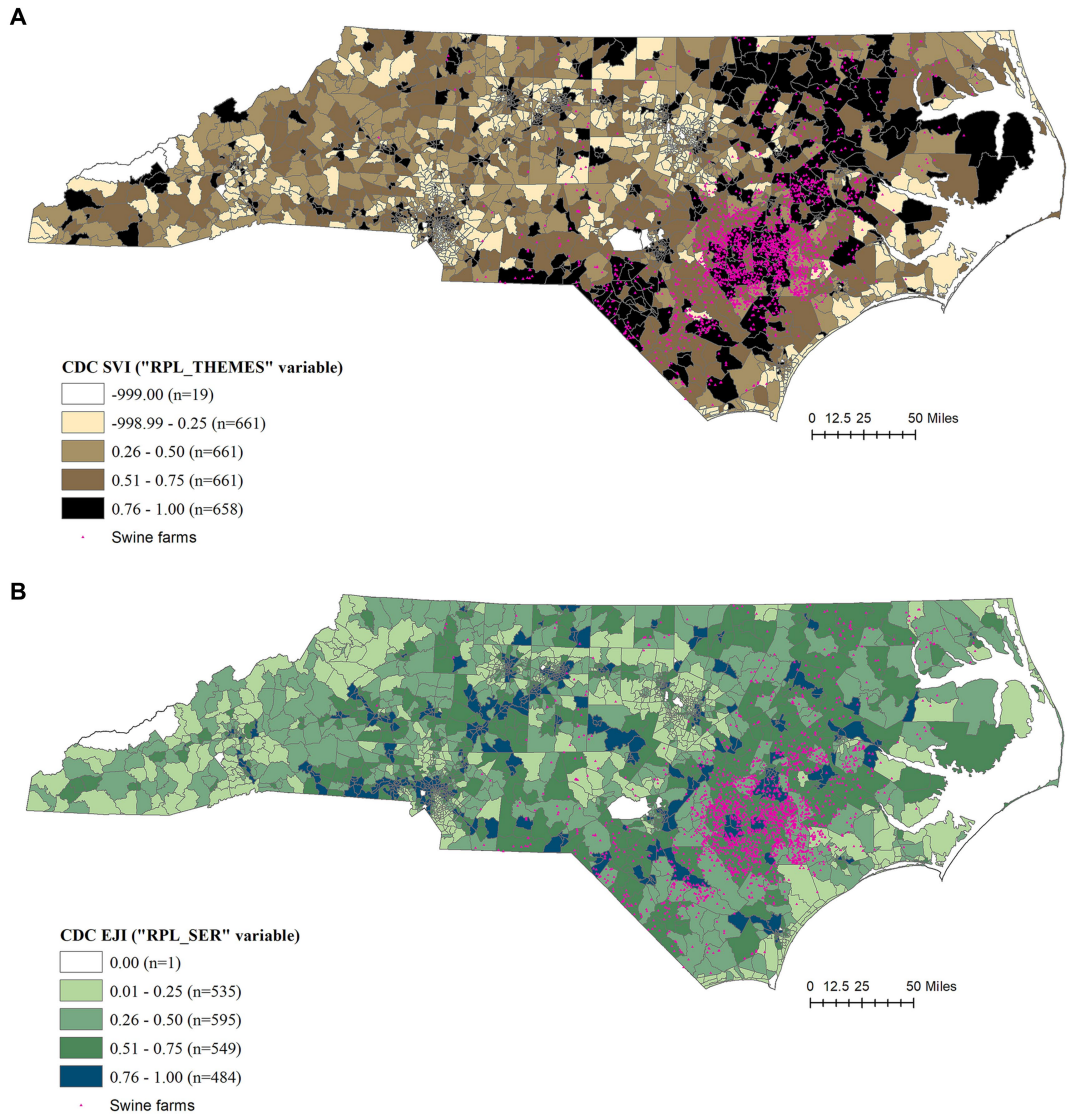
**(A)** Social Justice Index (SVI) developed by Centers for Disease Control and Prevention of the United States. The overall tract summary ranking of SVI represented by the variable (RPL_THEMES) from year 2022 index, available for 2,672 census tracts of North Carolina with 19 of the census tracts being ‘null’ values, was used in the analysis (7), (CDC SVI, 2022). **(B)** Environmental Justice Index (EJI) developed by Centers for Disease Control and Prevention of the United States. The census tract summary ranking of EJI excluding the health-related variables represented by the (RPL_SER) from year 2022 index, available for 2,192 census tracts in North Carolina with 28 of them being ‘null’ values, was used in the analysis (5), (CDC EJI, 2022).

Additionally, CDC SVI and EJI were mapped for visual comparison against the farming locations and the three indices developed here (Figures 2A,B). All indices were categorized and mapped using geometric intervals, with a separate group for “zero” values. The number of census tracts in each category is indicated in parentheses.

### Correlation

**Figure 3:**
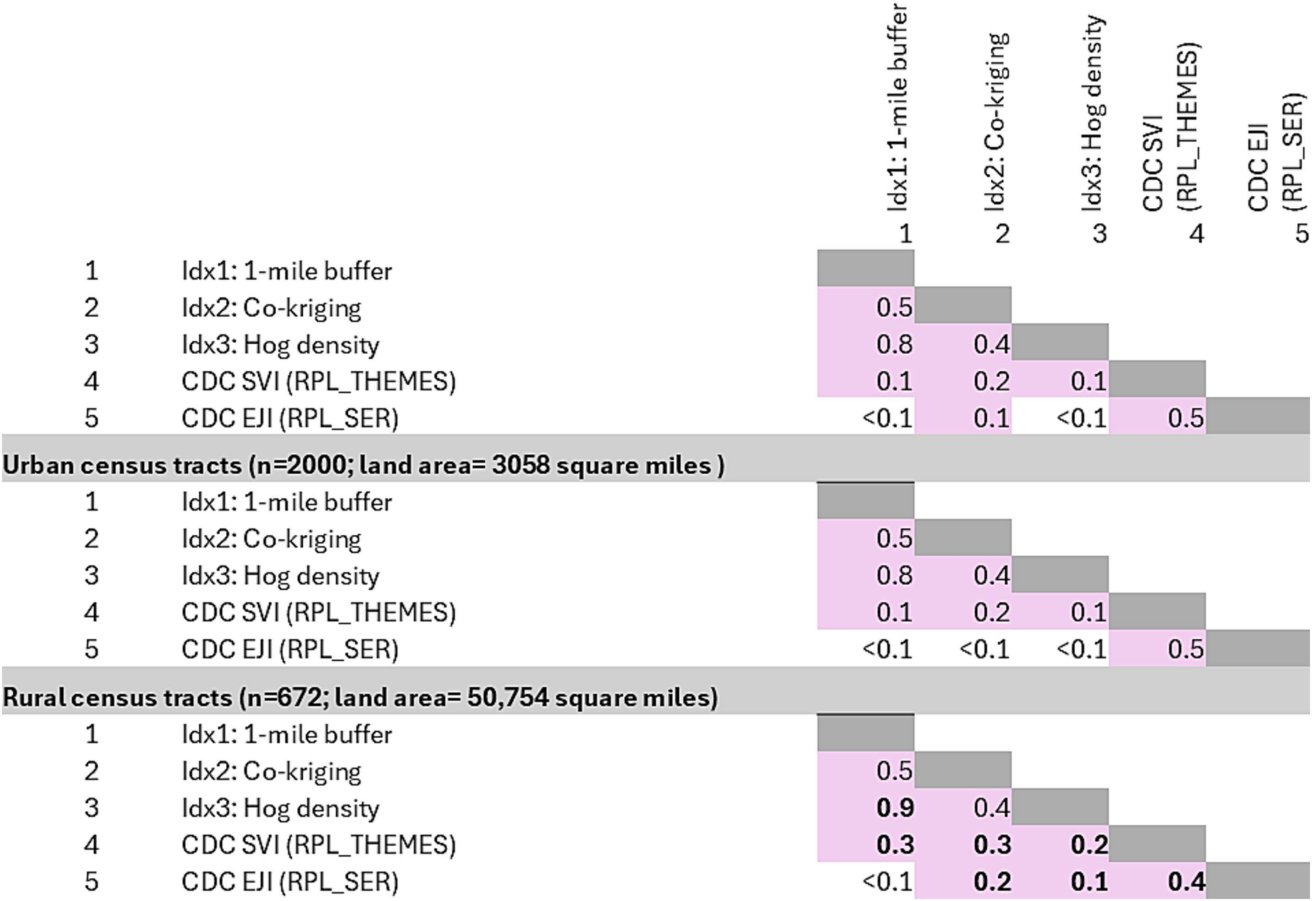
Spearman correlation coefficients were calculated to assess the relationships between three swine farm-specific spatial indices and the CDC’s Social Vulnerability Index (SVI) and Environmental Justice Index (EJI) at the census tract level for North Carolina, with separate analyses for urban and rural tracts. The correlation values that vary between urban and rural tracts are highlighted in bold.

All correlation coefficient values that were ≥0.1 at both urban and rural census tracts were summarized (Figure 3). Correlation values that differed between urban and rural tracts are highlighted in bold. At the overall state-level correlation analysis, the three indices differed visually, with IDx3 strongly correlated with IDx1 (0.8) and moderately correlated with IDx2 (0.4) (Figure 3). When compared between the urban and rural census tracts, the correlation between IDx3 representing hog density and IDx1 representing the number of addresses within 1-mile from a farm indicated a slight increase in the correlation coefficient from 0.8 to 0.9. CDC EJI and SVI were not prominently correlated with any of the three indices (0.1–0.3), however there was a slight increase in the correlation coefficient for rural areas compared to urban areas. As expected, SVI and EJI were correlated with each other at 0.5 as both these indices used compatible variables, with this correlation reducing to 0.4 at rural census tracts.

## Discussion

North Carolina, a state deeply rooted in agriculture, grapples with the intricate task of sustaining the vibrancy of its agricultural sector while concurrently addressing pressing concerns surrounding public health, environmental equity, and economic stability. We introduce three spatially based indices that quantify exposure to swine farming at the census tract level in North Carolina, to facilitate future exposure studies. Each index reflects a different definition and way of measuring the swine-farm specific exposure. Depending on the variables considered, the IDx2 which captures exposure to animal numbers and manure lagoons may have a better representation in exposure; followed by the IDx1 where addresses within 1-mile from a swine farm were quantified. A simple representation of hog density, as done in IDx3, may not fully capture the complexity of the issue compared to other methods of exposure estimation. However, the discrepancies between the maps and the varying correlations highlight how different methods of defining exposure can shape distinct narratives about which communities are most at risk. On the bright side, these exposure indices are relatively easy to develop on a yearly basis with updated farm data and can play a crucial role in policy relevance and developing sustainable solutions by providing clear, quantifiable metrics. This includes identifying high-risk areas, proposing data-driven policies, monitoring and assessment over time, and establishing early warning systems. Having clear and spatially explicit metrics that are specific to swine farms foster awareness encouraging multi-stakeholder engagement to design targeted and practical long-term solutions, while accounting for health and environmental risks.

In this study, the CDC’s Social Vulnerability Index (SVI) and Environmental Justice Index (EJI) were used as benchmark references to assess and compare the risks associated with swine-farm-specific indicators at the census tract level. These indices offer a standardized approach for evaluating social and environmental vulnerabilities, facilitating a comprehensive analysis of the potential health and environmental impacts of swine farming in relation to other contributing stressors. For example, the correlation coefficients between CDC SVI, EJI and the swine farm indices were weak, ranging from 0.2 to 0.3 in rural census tracts and even weaker in the urban census tracts (0.1–0.2) (Figure 3). Although these correlations are weak, they suggest that the socially and environmental vulnerable rural census tracts tend to have relatively more addresses within 1-mile from a swine farm (IDx1) or in areas where more animals and manure lagoons may also be located (IDx2).

EJI is proposed as an index to support public health officials, policymakers, and communities identify and address environmental and social factors affecting health, prioritize interventions, inform the public, analyze local impact drivers, and set measurable goals for advancing environmental justice and health equity (33). While mobile homes and exposure to agricultural pesticides by seasonal workers have been included in the CDC’s EJI, the index has placed less weight onto the animal agricultural aspects of exposure and has not included swine farming or animal farming specifically in the development process (5), (CDC EJI, 2022). As exemplified using the three spatial indices developed, the method of measuring exposure can lead to varying interpretations in relation to health and environmental outcomes. The low correlation of the swine farm-specific indices with CDC SVI and EJI shows the complex nature of the relationships when determining the direct exposure. A comprehensive approach is essential to quantify the cumulative impact of these contributors, prioritize their relative significance, and setting thresholds for intervention. This ensures protection of public health, environmental integrity, and the sustainability of farming industries and livelihoods dependent on them. Development of a hierarchical framework could help resolve conflicts while addressing social inequality, to enhance food security, production stability, and sustainability.

This ecological approach to developing spatial indices has several limitations that warrant consideration because identifying hazards and quantifying risks pose significant challenges (34). Firstly, by focusing on distance as a proxy for intensity of exposure, these indices may oversimplify the exposure, as the impact varies based on farm design and construction, as well as the specific water and soil characteristics of the farm’s location. Assigning a single value to a census tract can lead to inaccurate exposure estimates for specific communities, influenced by factors such as the Modifiable Areal Unit Problem (MAUP). This is particularly relevant in IDx1, where counts houses within a 1-mile radius of hog farms inevitably introduces such edge effects. While DEQ database provides the registered farm data for public usage, the permitted number of animals per farm does not accurately reflect the current or active animal population, as farm operations undergo turnover periods due to seasonal variations, breeding cycles, animal disease outbreaks, or other changes in management. Therefore, the interpretation of IDx2 and IDx3 should be made with this consideration in mind. Furthermore, having an index variable to reflect the manure management plan of the farm in the DEQ data would complement the available data analyses. In general, the developed spatial indices overlook individual-level exposure variations and confounding factors such as duration and route of exposure, including inhalation or ingestion. A more accurate approach would be a prospective, individual-level study over time and space, considering behavioral factors, to assess the causal health impacts of hog farm-related substances at both individual and neighborhood levels (35–37).

When conducting comprehensive exposure studies to establish dose–response relationships and estimate the magnitude, frequency, and duration of human exposure to swine farm-related agents—through direct measurement, scenario evaluation, or reconstruction—adherence to U.S. Environmental Protection Agency (EPA) guidelines is crucial (29, 38, 39). Investigating health issues in communities near swine farms is challenging, especially defining exposure, which involves capturing proximity, duration, frequency, population vulnerability, and cumulative environmental impacts for comparison. Moreover, when developing or using such spatial-indices as indicators of exposure leading to certain health outcomes, we should also consider common structural factors—like political, financial, and social status—that drive health inequities. Understanding the nuances and defining exposure accordingly is essential for addressing this multifaceted issue affecting humans, animals, and their shared environment.

It is crucial to recognize that minority communities, often disproportionately impacted by environmental injustices, were not deliberately targeted by animal or crop agriculture (37, 40). Especially for North Carolina, the issue inseparable from the state’s historic background led to farm establishments in these neighborhoods and finding ways for balancing the state’s agricultural heritage with the imperative of environmental justice requires a multifaceted approach. To tackle these challenges, state governments and stakeholders have various avenues to explore including enforcing stricter regulations and ranking and monitoring practices for all environmental issue contributors (10, 20, 21, 41). These solutions may include policy reforms offering incentives for transitioning from traditional to sustainable practices, such as tax breaks, subsidies, and grants, or imposing environmental regulations to ensure compliance with sustainable farming practices. Regulations alone would not bring the farming communities to act upon costly changes to the facilities and their waste disposal plans. Some of these changes require substantial investments in costs, labor, time, and technology (42, 43). Animal farming as an industry has its own fair share of challenges to overcome including foreign animal diseases crossing borders, climate effects on animal health, detrimental impacts of endemic diseases, and issues related to bringing in seasonal farm labor (44). The farming communities require comprehensive cost-effective and cost–benefit analyses before implementing any changes and often need technical assistance from the state and federal agencies to effectively carryout the necessary and sustainable management adjustments.

Environmental stewardship is pivotal in promoting the wellbeing of both farming and minority neighboring communities. This necessitates adopting a true One Health approach, which convenes stakeholders from government, farming industry, and communities to address the complexities of optimizing animal, human, and environmental health, all while promoting sustainable agriculture to fulfill the nation’s food production and economic requirements. In the meantime, monitoring of air, water, and soil qualities is imperative to determine the health of the environment and its future trajectory. Establishment of buffer zones between farms and residential areas or the use of spatial indices to identify census tracts requiring stringent monitoring protocols can be proposed to help both farming communities and neighboring residents better understand the status quo and mitigate the potential health and environmental impacts.

## Conclusion

A place-based assessment of exposure, along with initiatives to address environmental justice issues and strategies to improve access to healthy air, sustainable land, clean water, and a better climate is a multifaceted and challenging task for all stakeholders, governments, and policy makers. The three novel spatial indices quantifying exposure to swine farming introduced here underscore the potential of exposure-specific indices in advancing both health research and sustainable agriculture. However, the process of defining and measuring exposure, particularly with factors such as proximity, duration, frequency, vulnerability, and cumulative impacts, is complex and challenging. This study underscores the importance of establishing a hierarchical framework to accurately quantify and compare environmental exposures, considering risk-modifying factors and individual-level exposure across both space and time, prior to drawing conclusions about direct exposure risks.

It is crucial to develop a hierarchical framework for quantifying the comparative magnitude of various environmental exposures, using pollutant-specific exposure indices tailored to local realities in order to gain nuanced insights and support informed decision-making in agricultural practices.

## Data Availability

The original contributions presented in this study, derived from publicly available sources, are adequately cited in the article and/or Supplementary material. Further inquiries can be directed to the corresponding author.

## Footnotes

^1^https://www.nconemap.gov/; all accessed by 20 June 2023

^2^ https://deq.nc.gov/

^3^ https://waterwatch.usgs.gov
