# Supplementary material for: Spatial indices quantifying exposure to swine farming in North Carolina
Source: Front Vet Sci. 2025 Apr 30;12:1552028. doi: 10.3389/fvets.2025.1552028 (PMC12075876; doi:10.3389/fvets.2025.1552028)
Supplement: Supplementary file 1 [file Table_1.docx]

# **Supplementary material**

**Supplementary Table S1.** Parameters used in the co-kriging model^*^.

| **Parameter** | **Setting** | |
| --- | --- | --- |
| Lag size | 0.007 | |
| Number of lags | 12 | |
| Models | Exponential | |
| Major range | 0.06 | |
| Nugget | 0 | |
| Search neighborhood parameters for each predictor variable | Primary variable:  Log of number of animals permitted per farm location | Secondary variable:  Number of manure lagoons belong to farm location |
| Minimum number of neighbors to be included in the kriging process | 5 | 5 |
| Maximum number of neighbors to be included in the kriging process | 2 | 2 |
| Sector type | Four and 45 degreee | Four and 45 degreee |
| Angle | 0 | 0 |
| Anisotropy factor | No | No |
| Root Mean Square value (RMS) of the model | 0.057 | |
| RMSE standardized value of the model | 3.86 | |

^*^Understanding cokriging: <https://desktop.arcgis.com/en/arcmap/latest/extensions/geostatistical-analyst/understanding-cokriging.htm>
